# Ecosystem activation system (EAS) technology for remediation of eutrophic freshwater

**DOI:** 10.1038/s41598-017-04306-3

**Published:** 2017-07-06

**Authors:** Xiaoli Chai, Boran Wu, Zhongshuo Xu, Ning Yang, Liyan Song, Jingjing Mai, Yang Chen, Xiaohu Dai

**Affiliations:** 1 State Key Laboratory of Pollution Control and Resource Reuse, College of Environmental Science and Engineering, Tongji University, 1239 Siping Road, Shanghai, 200092 China; 2 Environmental Microbiology and Ecology Research Center, Chongqing Institute of Green and Intelligent Technology, Chinese Academy of Science (CAS), 266 Fangzheng Avenue, Chongqing, 400714 China

## Abstract

Ecosystem activation system (EAS) was developed to create beneficial conditions for microbiome recovery and then restore and maintain the ecological integrity (microbial community, phytoplankton, zooplankton) for eutrophic freshwater rehabilitation. A 30 day’s filed test of EAS indicated that over 50% of contaminant was removed and the algae visibly disappeared. EAS treatment 2.5-fold increased the diversity of microbial community and changed the microbial community structure (e.g., two and three-fold decrease in the amount of *Flavobacterium* and *Pseudomonas*, typical abundant species of eutrophic freshwater, respectively). Further, the diversity of phytoplankton and zooplankton of treated water suggested that these species were diverse. Representative phytoplankton of eutrophic freshwater, *Chlorella* and *Chlamydomonas* were undetectable. The possible mechanism of EAS is restoring the trophic levels of the water body via bottom-up approach by microbial community.

## Introduction

The deterioration of fresh water quality resulting from nitrogen, phosphorus and organic pollutant enrichments is a major challenge for water remediation in China^1^. Most lakes (e.g., Lake Taihu, Lake Dianchi, and Lake Chaohu) suffer from water quality deterioration as a consequence of economic development^2^. Of 968 monitoring stations on 423 major rivers and 62 key lakes (or reservoirs), only 3.4% of the stations had class I water quality while 30.4% had class II water quality according to the Chinese national standards for surface water^3^. For example, a cyanobacteria bloom occurred in Lake Taihu in 2007 resulting in a domestic water shortage for 5 million people in Wuxi, Jiangsu Province^2^.

Conventional activated sludge treatment is the most widely used technology to remove the contamination (e.g., chemical oxygen demand (COD), total nitrogen (TN) and total phosphorus (TP)) of domestic sewage via microbial metabolism. However, the activated sludge method is unsuitable for *in situ* rehabilitation of natural bodies of water due to limited treatment capacity, complicated facility construction, high energy consumption (1.0–1.5 kWh/m^3^)^4, 5^, and the required drainage system. In addition, activated sludge technology produces a large amount of excess sludge that needs further treatment.

Many technologies have been used for *in situ* rehabilitation of natural water bodies. These include physical and chemical methods (e.g., sewage interception, sediment dredging^6^, exchanges with fresh water, bypass treatment^7^, artificial aeration^8^, and biological methods such as phytoremediation^9^. However, these methods are often unsuitable due to disadvantages such as costs and the secondary transfer of pollutants. For example, sewage interception is very expensive and also significantly increases pressures on the capacity of urban sewage systems and wastewater treatment plants. Deep dredging damages the ecology of river bottoms, while shallow dredging does not eliminate the pollutants. Phytoremediation e.g., constructed wetlands (CWs), hydroponic nutrient film technique (NFT) systems, and floating mat economic plant-based treatment systems (FMETSs) were once thought to be reliable and economically viable technologies for remedying eutrophic water^10^. However, the nutrient removal efficiency is strongly dependent on local climate conditions (temperature, precipitation, and sunlight) which can limit widespread and long-term applications^11–13^. Artificial aeration can improve water quality in a short period by increasing dissolved oxygen (DO) to enhance the biological oxidation of organic pollutants, but the large energy consumption limits its wide application. Water transfer is also a feasible method to improve water quality, however, it can’t restore the self-purification capacities of water ecosystem. Therefore, innovative technology is urgently needed for *in situ* rehabilitation of polluted natural bodies of water.

Increasing the collection and treatment ratio of wastewater and upgrading the discharge standards of wastewater treatment plants are both good strategies for reducing water pollution. These approaches decrease the pollution input and allow the water ecosystem time to recover biological function and purity. The end result is significantly improved water quality in those water bodies that were previously severely polluted. Developed countries, e.g. Germany, United States of America and so on, have used this strategy to deal with polluted water during industrialization, and now the water quality has greatly improved. This strategy is effective for water body recovery, but it requires long-term effort (>10 years). It is less feasible for China and other developing countries to purify polluted water bodies via this time-consuming strategy because the rapid economic development conflicts with it. However, the urgent need for high quality water requires innovative technology for rapidly, and efficiently, dealing with the problems of polluted water.

**Figure 1:**
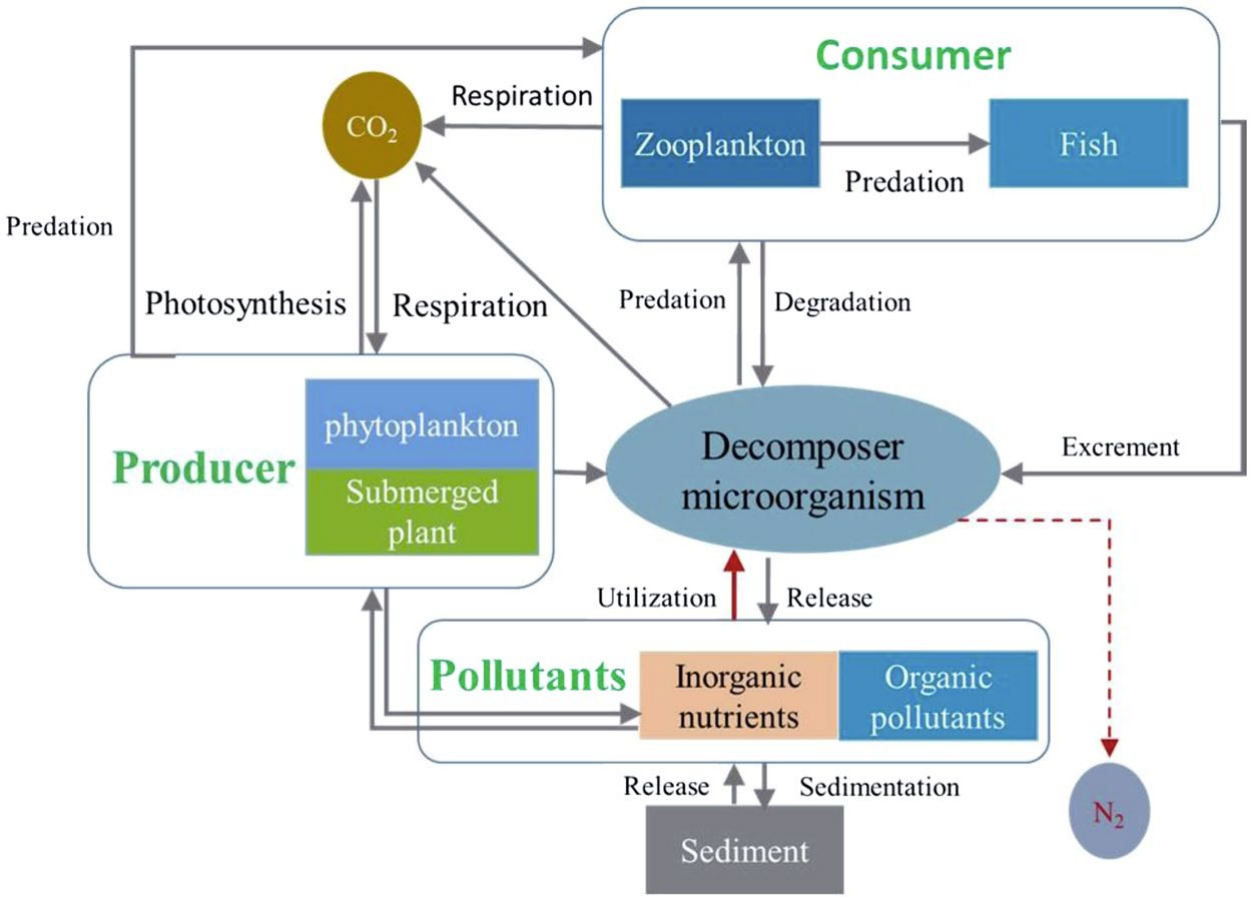
Proposed mechanism of EAS.

We developed an ecosystem activation system (EAS), based on the principle of water self-purification, to create favorable conditions for restoring the self-purification functions of polluted water ecosystems. We simulated the contaminant removal function(s) of the microbial community in the activated sludge process and hypothesized that the fresh water microbial community also plays a key role in water body self-purification. The microbial community is central in material transformation (production/decomposition) from lower trophic levels to higher trophic levels, including submerged plant, phytoplankton, zooplankton, and aquatic animals (Fig. 1). Through reconstructing and extending the food chain of a natural water body by enhancing the key functions of the microbial community, the ecological function of the polluted water body can be restored, and water quality can be improved.

EAS technology has 3 reaction stages: anoxic, aerobic, and releasing. First, the polluted water is pumped into EAS, and microorganisms that are beneficial for water quality improvement (nitrobacterium, denitrifiers, COD decomposers, and phosphate-accumulating organisms) are activated by the addition of a specific activating reagent. Second, the biomass of these beneficial species can increase exponentially relying on the added specific activating reagent. Third, the beneficial microorganisms activated by the system were released into the water body with effluent. As a result, the microbial community structure of the water ecosystem was continuously restored. The food chain of the restored ecosystem was re-established based on the metabolism changes of microbial community and the self-purification function of the restored water ecosystem. The process led to purification of the water system.

## Results and Discussion

### Technical principle of EAS

**Figure 2:**
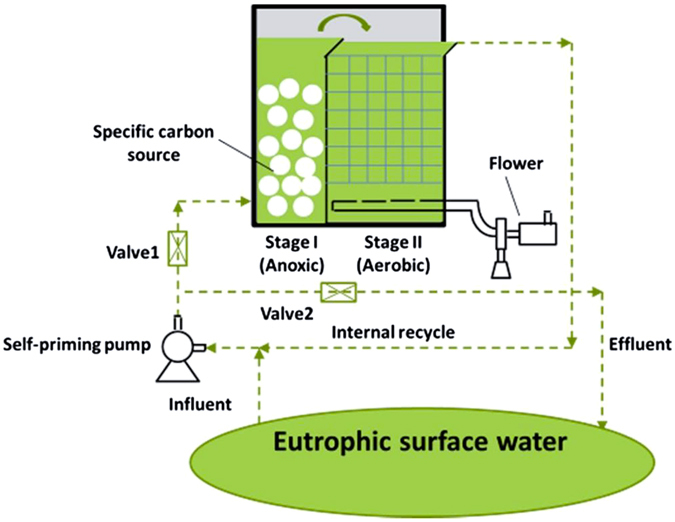
Constituents of EAS.

A pilot-scale open-mouthed biological filter bed was constructed as the activation system for the water ecosystem. The EAS was composed of an anoxic stage and an aerobic stage (Fig. 2). The ratio, by volume, of the anoxic to the aerobic stage was 1:5. The total volume of the reactor was 0.6 m^3^. The anoxic stage was operated as an open-mouthed biological filter bed. Polyhydroxyalkanoate (PHA) can not only serve as easy-consuming carbon source for microorganism but also the specific activating reagent only for nitrifying-denitrifying bacteria and phosphorus-accumulating bacteria in conditions where exogenous N and P are lacking^14–16^. If PHA is artificially added to the water, it can specifically favor the reproduction of whole microbial community and beneficial nitrifying-denitrifying bacteria and P-accumulating bacteria^17^. In this study, PHA particles, with a size range of 5–10 mm, were chosen both as biofilm carriers in the anoxic stage and as an activating reagent for the promotion of the growth of the microbial community with specific physiological functions, including nitrifying and denitrifying, and phosphorus accumulation based on previous test results^14–17^. The fill amount of PHA particles was 2/3 the volume of the anoxic stage. Hence, the extended anoxic stage, operated as a moving bed biofilm reactor with PHA particle carriers, could enhance the reproduction of the specific desirable microorganisms. After massive proliferation, the specific desirables microorganisms were released into the water to improve the N- and P-removal capacity of the microbial system.

The aerobic stage was established after the anoxic stage to enhance the oxidation of ammonia to nitrate and for P uptake. A polyurethane filler was applied as the filling material of aerobic stage and the filling amount was 2/3 of the aerobic volume. P uptake was performed by the specific microorganisms activated by PHA in the anoxic stage. Accordingly, the microorganisms with P accumulation are released into the water body and involved in metabolic process of organism. The P would then be metabolized through the entire material flow of the ecosystem. The aerobic stage was also set up to enhance utilization of the natural dissolved organic matter (DOM). Thus, the reproduction of competitive species that depend on the natural DOM instead of PHA would be inhibited with the depletion of carbon source. A self-priming pump was used to balance influent, effluent and inner recycling of EAS.

EAS was used in a typical urban freshwater body (area: 8000 m^2^; average depth: 2.5 m) with high degree of eutrophication. After 30 d of operation, the eutrophication bloom was completely removed and the water quality was significantly improved.

### Specific features of EAS

The EAS design achieved highly efficient culture and activation of the desirable microorganisms in continuous flow. Influent, effluent, and the inner cycle were all moved by a single self-priming pump. Through adjusting the flow rate of influent, effluent and the inner cycle, the mass balance and steady operation of the system were achieved. The design goal of the inner cycle was to provide sufficient cultivation time for the activated dominant bacteria and to maintain the biomass retention in the reactor. The suspended polyurethane carrier in the aerobic stage had a large specific surface area (11.5 m^2^/g) offering beneficial conditions for microbial attachment and improving the functional biomass. The pore structure inside and outside the suspended polyurethane carriers also created both anaerobic and aerobic conditions at the micro scale, allowing for simultaneous nitrification and denitrification. The reactor had simple operation protocols. The energy consumption of both pumps and mechanical agitation during the anoxic stage was <600 W per system run because the large influent flux and large aeration intensity required by conventional bypass wastewater treatment are avoided.

### Water quality improvement

#### Sensory quality index of the test water body

Reducing the unpleasant odor and chroma (color) and rehabilitating the water ecosystem function were the major goals of this pilot-scale experiment. At the beginning of the EAS operation process, the surface of the water was densely covered by suspended algae clouds and was blackish green, seriously limiting water clarity. After 15 d of treatment, the sensory quality index was considerably improved resulting from a decline in suspended algae and the water being clear to the bottom (Fig. S1). Accordingly, the turbidity and chroma values significantly decreased from initial 20 NTU to 5.8 NTU and 24 PCU to 10.667 PCU, respectively (Fig. S1 of Supporting Information). During the follow-up 75-d treatment, no obvious algae were found, the turbidity and chroma values continued to decrease, and the water body remained clear.

#### General water quality of the test water body

**Figure 3:**
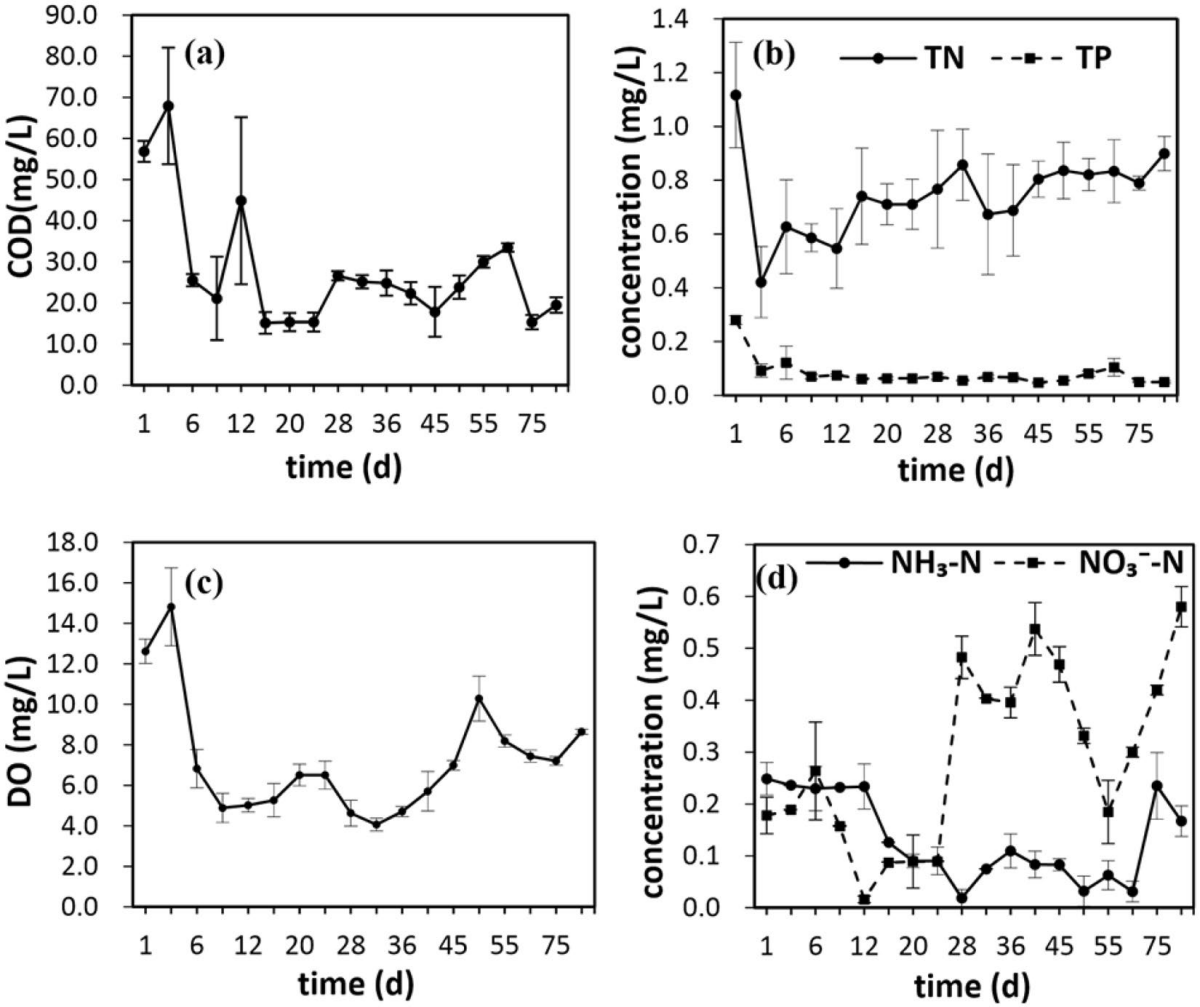
Variation of water quality (**a**: COD; **b**: TN and TP; **c**: DO; **d**: Ammonia and nitrate) of study water body during 90 days’ EAS remediation.

Similar to the decline of suspended algae, the main water quality index, TN, TP, and COD also continuously decreased (Fig. 3). Reduction of TN, TP, and COD could be realized in only 3–15 d after initiating the EAS operation. The removal efficiencies of these 3 pollution indexes were all >50% (77.7% for COD, 53.5% for TP, and 53.2% for TN). These results indicate that EAS efficiently reduced the level of nutrients.

Short-term increases in TN, TP, and COD occurred on days 5, 12, 28, and 62. This is probably because of external contaminants introduced via rainfall on these days. Each individual precipitation events totaled around 3–5 mm. Notably, the elevated TN, TP, and COD levels decreased within the following few days. The contaminant disturbance by rainfall and the subsequent rapid removal of the main contaminants (TN, TP, and COD) indicated that the water body had active self-purification and self-regulatory functions. The reorganized microbial community can extend the material flow of the degradation of main contaminants and enhance the transformation of specific contaminants from lower trophic levels to higher trophic levels by decomposers. In this case, the pollutants and the corresponding metabolic products were spread among the trophic levels of the ecosystem, and the capacity for resisting impact loads was restored. For example, in a complete and healthy water ecosystem, the predation process will play an important role in the transferring N & P from the low trophic levels, the microbial metabolism of nutrients, to the high trophic levels, such as zooplanktons, aquatic plants, fish and so on. The measured decrease of P could be mainly due to the dilution effect through the whole food chain of the water ecosystem.

A decreasing level of eutrophication in the water body was confirmed by DO variation during the operation periods. The value of DO decreased during the first 10 d of operation, possibly resulting from oxygen consumption due to decomposition of dead algae. The initial DO decrease was also due to photosynthesis reduction of suspended algae, which corresponded to the reduction of COD in water body^18, 19^. A subsequent increase was linked to the reduction of organic contaminants resulting in the decline of heterotrophic microorganisms.

Modification of the N cycle and the removal of dissolved P are the key to successful rehabilitation of eutrophic water bodies because excess N and P input induces algae growth^9, 18^. In this research, the ammonia concentration decreased during the operation period. Accordingly, the nitrate and total nitrogen increased, suggesting nitrification function development in the water body.

#### Water quality improvement evaluation

The water quality parameters of the raw and treated water are listed in Table S1. The concentrations of TN and COD in the raw water body were approximately 1.2 and 3 times higher, respectively, than the class II reference values for surface water quality (according to Chinese national standard, GB3838). Pollution of the surface water was severe and it had dense algal growth. As a result, the chroma and turbidity values considerably exceeded the standards. The water body was blackish-green color and had an unpleasant odor. After a 30-d operation of EAS, the transparency of the surface water improved considerably, and the blackish-green color disappeared. All of the water quality parameters then met the standard class III values for drinking water sources according to GB3838.

As an easy-consuming carbon source for microorganism, PHA can induce the growth of the whole microbial community, including specific groups (nitrifying and denitrifying bacteria, and phosphorus accumulation bacteria). Accordingly, enhancement of metabolism of microorganism consumed more carbon, nitrogen, phosphorus, and other elements. This will lead to the improvement of water quality. The improved water quality would positively impact zooplankton, phytoplankton and aquatic plants in water ecosystem. In a healthy water ecosystem, the predation process plays an important role in the transferring N & P from the low trophic levels to the high trophic levels. All trophic levels and microbial community metabolism accounted for the decrease of P. Interestingly, large amount of advanced plants (i.e. *Bryophyte*) appeared on the surface of underwater rocks (Fig. S1d) after 30 days’ EAS operation, suggesting N and P are essential nutrients for whole microbial community and food chains. Therefore, the dominant P distribution has been extended to the higher trophic levels, and support the growth of aquatic plants, instead of leading to the algae bloom.

### Sediments physiochemical parameters variation

Sediment is an indispensable part of substance circulation in water ecosystems. The concentrations of TN and TP decreased from 0.23 wt. % dry solid (DS)  to 0.13 wt. % DS and 0.37 wt. % DS to 0.21 wt. % DS, respectively, and this helped to limit the growth of algae (Table S2). The decrease of VS indicated that organic pollution was reduced in the sediment and the material cycle from sediments to the aquatic phase or the atmosphere was enhanced in the EAS treated water body.

### Microbial community diversity

The sequence composition analyses showed that the bacterial diversity estimates for the EAS treated water were much greater than raw water diversities. This is strong evidence for microbial community shift in the water body. The mean number of ACE for EAS treated samples was 8607 ± 944, which was greater than that found in raw water (3488 ± 374). Similar, the other bacterial diversity measures (Chao and Simpson) of EAS treated water were much higher than those of untreated water (Table 1).

**Table 1:** Comparison of diversity index of microbial community in study water body before and after 30 days’ EAS remediation.

		ACE	Chao1	Shannon	Simpson	Goods Coverage
Raw	S1	3701.4	3548.0	6.6	0.95	0.99
Water	S2	3726.4	3536.3	6.0	0.95	0.99
	S3	3589.5	3476.4	7.1	0.97	0.99
	S4	2934.9	2683.0	4.4	0.87	0.99
	Average	**3488.1**	**3310.9**	**6.0**	0.93	0.99
	SD	373.5	419.8	1.2	0.05	0.00
EAS	S1	9157.7	8625.1	8.1	0.98	0.97
Treatment	S2	9559.8	8990.6	8.1	0.98	0.98
Water	S3	8261.3	7642.9	7.6	0.97	0.97
	S4	7449.1	7027.8	7.4	0.95	0.98
	Average	**8607.0**	**8071.6**	**7.8**	0.97	0.97
	SD	943.6	899.0	0.4	0.01	0.01

**Figure 4:**
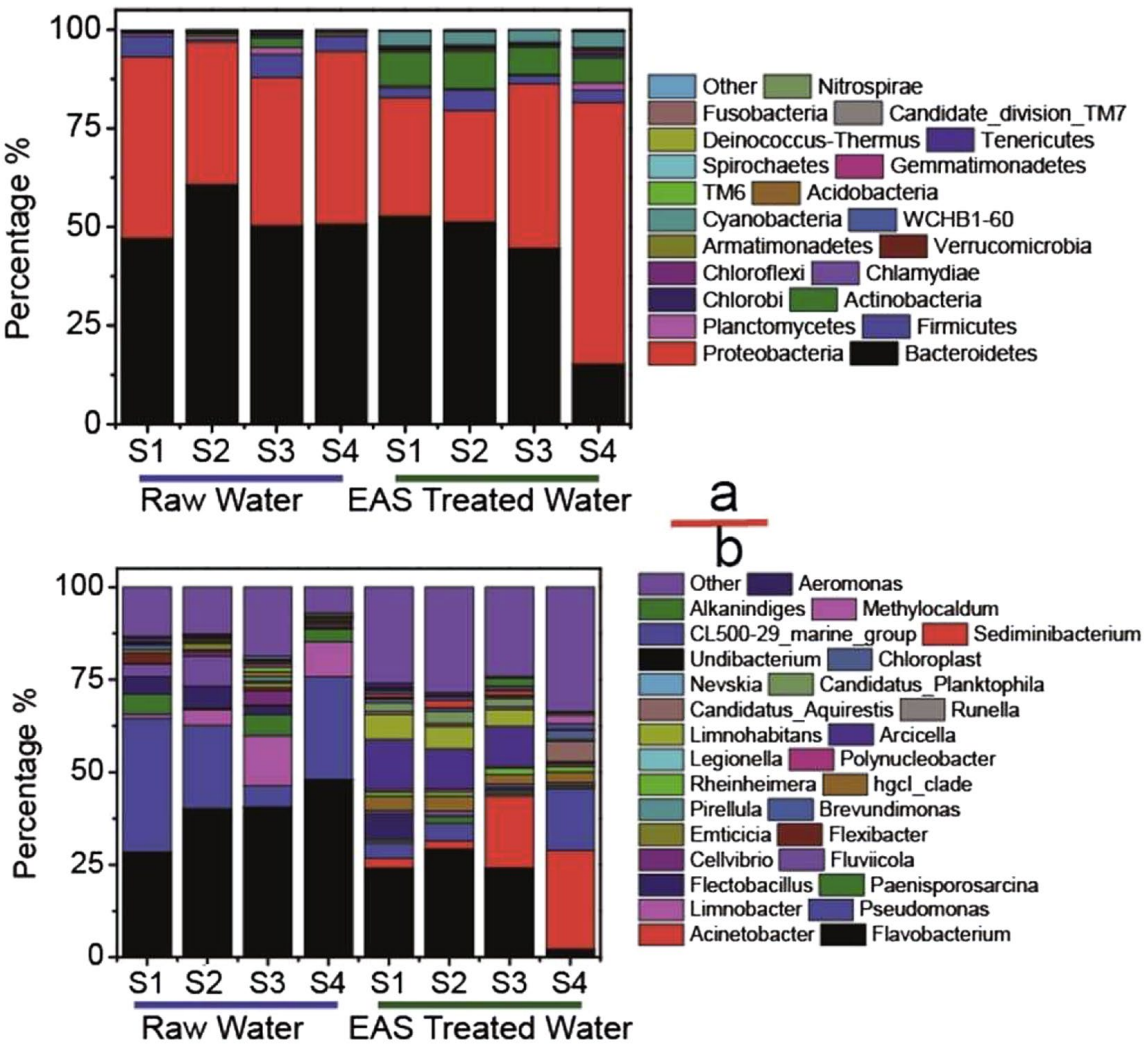
Comparison of microbial community structure (phylum level: **a**; genus level: **b**) in study water body before and after 30 days’ EAS remediation.

For the samples of raw water (4 samples) and EAS-treated water (4 samples), the most commonly detected phyla were *Proteobacteria*, *Firmicutes*, *Bacteroidetes*, and *Acidobacteria* (Fig. 4a). For the 4 samples of raw water, *Proteobacteria* (36.2% to 46.0%), *Bacteroidetes* (47.2% to 60.8%), and *Firmicutes* (0.5% to 5.4%) were the most dominant groups. In contrast, *Proteobacteria* (28.1% to 66.4%), *Bacteroidetes* (15.3% to 52.8%), and *Acidobacteria* (6.4% to 9.5%) were the dominant representatives of EAS treated water.

In order to further compare the difference of bacterial communities in raw water and EAS-treated water, it is preferable to analyze the sequencing date at the subdivision level. The bacterial communities at the genus level are presented in Fig. 4b. Generally, *Flavobacterium* (affiliated with *Bacteroidetes*) and *Pseudomonas* (affiliated with *Proteobacteria*) were predominant at all 8 samples, representing 2.2% to 48.1% and 0.7% to 36.1% of the total genera. However, the relative abundance of *Flavobacterium* decreased from 39.4 ± 8.1% to 20.0 ± 12.1% and *Pseudomonas* decreased from 23.0 ± 12.9% to 6.6 ± 6.9% after EAS treatment. Similarly, *Limnobacter* (affiliated with *Proteobacteria*) diminished 7.1 ± 5.5% in untreated water. *Flavobacterium* and *Pseudomonas* are the common bacilli that cause pneumonia, meningitis, and sepsis^20, 21^. After 30 d of EAS treatment, the relative abundance of *Flavobacterium* and *Pseudomonas* decreased along with the disappearance of algae, suggesting *Flavobacterium* and *Pseudomonas* play a role in the control of algae blooms.

Nevertheless, it can be found that the sequence affiliated to *Acinetobacter* (affiliated with *Proteobacteria*) was not detected in raw water (4 samples), whereas the corresponding data in EAS-treated water accounted for 2.51%, 2.08%, 19.44% and 26.69% (average 12.7 ± 12.4%) of total bacterial sequences, respectively. The bacterial sequences affiliated to the genus *Arcicella* from the four untreated water samples were only 0.88%, 0.18%, 0.06% and 0%, while in the four EAS-treated water samples the sequences closely related to *Arcicella* accounted for 13.62%, 10.83%, 10.71 and 0.43% (average 8.9 ± 5.8%) of the total biomass, respectively. Meanwhile, the abundances of the genus *Limnohabitans* in the four raw water samples were 0.01%, 0.08%, 0.22% and 0.66%, whereas those in four EAS-treated water samples were 6.64%, 5.97%, 4.6% and 0.33% (average 4.39 ± 2.83%). At the same time, the relative abundance of more planktonic bacteria, usually dwelling in healthy freshwater, increased. These bacteria including *Candidatus Planktophila* (from 0.2 ± 0.1% in untreated water to 2.0 ± 2.3% in EAS treated water) and *Candidatus Aquirestis* (from 0% to 2.1 ± 1.1%) can be regarded as positive indicators of water quality improvement^21, 22^. Interestingly, some functional bacteria involved in the C and N cycles, such as *Methylocaldum* (from 0% to 0.9 ± 0.9%), *Undibacterium* (from 0% to 0.2 ± 0.1%), and *Chloroplast* (from 0% to 1.3 ± 0.9%), appeared. These results indicated that ecosystem activation system (EAS) technology increased the relative abundances of the key functional microorganisms *Acinetobacter*, *Arcicella* and *Limnohabitans* in the EAS-treated water.

*Acinetobacter* plays a significant role in phosphorus removal^22, 23^ and has been detected in various biological phosphorus removal reactors and other environmental samples^24^. Wagner, M. *et al*.^24^ evaluated the dynamics of phosphorus accumulating organisms (PAOs) in their activated sludge system for biological nutrients removal and found that the populations of PAOs was mainly constituted by the bacterial genera of *Acinetobacter* sp., *Bacillus* sp. genera etc., with *Acinetobacter* being dominant in the entire communities. In this study, abundant *Acinetobacter* occurred in EAS treated water, accounting for the decrease of phosphorus concentration owing to its phosphorus utilization.

*Limnohabitans* (affiliated with *Beta*-*Proteobacteria*) plays a prominent role in the nutrients cycle of freshwater^25, 26^. Interestingly, *Limnohabitans* can uptake substrate in high rates, be able to grow on algal-derived substrates and has high mortality rates. Combination of these provides them with a strong competitive ability of nutrients. The average abundance of *Limnohabitans* in the EAS treated water was 18 times higher than that of raw water, which also benefited the decrease of the nutrient concentration in the ecosystem.

In the typical eutrophic lake, the microbial community depends on variability of environmental conditions nutrient levels. Under extreme conditions of poor nutrition, the diversity of microbial communities is reduced^23^. With improvement of nutritional conditions, the species of microorganism increases. In the case of ultra-eutrophication or an extremely eutrophic lake, the microbial community diversity is reduced due to the high nutrient load. In addition, the release of algal toxins by the lysis of microcystis can limit the growth and reproduction of microorganisms^23, 25^. In this study, PHA was added to promote the growth of nitrifying-denitrifying bacteria and phosphorus-accumulating bacteria. Illumina sequencing gave us whole microbial community structure including the specific nitrifying-denitrifying bacteria and phosphorus-accumulating bacteria. It was found that EAS technology substantially increased the relative abundances of the key functional microorganisms *Acinetobacter*, *Arcicella* and *Limnohabitans*, which played significant roles in phosphorous accumulating and resulted in the decrease of dissolved TP in the EAS-treated water. As a result, the concentrations of TN and TP in the water and sediments decreased significantly, suggesting that N and P were greatly consumed by microorganisms. At the same time, the accumulation of phosphorous by microbial communities would not lead to the increase of suspended solid causing the turbidity of the water body. Furthermore, the submerged plants grew abundantly with the improved transparency of water body, so that the toxic substances can be accumulated in the submerged plants^26^. This further improved conditions beneficial for growth of the microbial community.

### Phytoplankton variation

After 30 d of treatment, the water quality was significantly improved and remained stable. Thus, 7 phyla (57 genera) of phytoplankton were detected based on the water samples at 30 d (Table S3). There were 25, 13, 12, 3, 2, 1, and 1 genera belonging to *Chlorophyta*, *Euglenophyta*, *Bacillariophyta*, *Cryptophyta*, *Pyrrophyta*, *Xanthophyta* and *Chrysophyta*, respectively. The average concentration of phytoplankton was 149.87 × 10^4 ^L^−1^, ranging from 126.32 × 10^4 ^L^−1^ to 179.07 × 10^4 ^L^−1^. *Cryptophyta* had the largest proportion of density specific gravity of 49.6%, followed by *Chlorophyta* (40.6%) and *Bacillariophyta* (8.2%), respectively.

The number of dominant species plays an important role in community structure stability^27^. The community structure is more intricate and stable if the dominant species are more diverse^28^. As shown in Table S4, 6 dominant species: *Chroomonas acuta, Pediastrum simplex* v. *duodenarium, Cryptomonas ovata, Nitzschia* sp., *Scenedesmus quadricauda*, and *Cry. erosa* were dominant.

*Chlorella* is usually the most abundant and seasonally stable genus in eutrophic lakes with a concentration range of 1–5 × 10^6^ L^−1^ ^29–32^. *Chlamydomonas* is also a representative phytoplankton of lakes with accumulated N and P nutrients, with concentrations of 10^2^–10^3^ L^−1^ ^29^. However, *Chlorella* and *Chlamydomonas* were not detected in the rehabilitated water body in this study. Another species, *Chlorophyta*, usually has a density of 2 × 10^7^ L^−1^ in eutrophic freshwater^33, 34^. In this study, its density ranged from 41.77 × 10^4^ to 74.76 × 10^4^ L^−1^, which was 2 magnitudes lower than levels in eutrophic freshwater. Comparison of abundances of typical phytoplankton species in eutrophic freshwater and in the rehabilitated freshwater of this study indicated that eutrophication of EAS treated freshwater was inhibited. This is also indirectly reflected by the variation on turbidity before (NTU 20) and after (NTU 5.8) treatment.

The diversity indices of phytoplankton are shown in Table 2. The value of H’ in this study was 2.34, which was higher than values of water bodies with poor water quality (1.5–2.0). The Margalef richness index of eutrophic water bodies usually ranges from 0–0.5^35^. In this case, the value of d was 1.37, which is almost 3 times higher than that of eutrophic water bodies. In addition, the value of D (3.3) in this study was also in the range for good diversity (3.0–6.0) and much higher than the typical value (0.2–0.5) of eutrophic lake documented in other reports^35–39^. The values of above 3 indexes indicate that the EAS treated freshwater body has good phytoplankton diversity.

**Table 2:** Ecological index of phytoplankton and zooplankton community in study water body after 30 days’ EAS remediation.

Index	Max	Min	Average
*H*′ phytoplankton	2.48	2.18	2.34
*d*	1.43	1.27	1.37
*D*	3.53	2.98	3.30
*H*′ zooplankton	1.49	1.40	1.43
*d*	0.33	0.28	0.30
*D*	2.41	1.89	2.09

### Zooplankton variation

Zooplankton is another important link of pollutant cycles in the water ecosystem. A total of 6 species of zooplankton were detected; 4 species of *copepods* and 2 species of *cladocera*. The average concentration was 2.66 × 10^4^ m^−3^ with a range of 1.89–3.74 × 10^4^ m^−3^. The dominant zooplankton species were *Harpacticoida* and *Bosmina sp*. The diversity index of zooplankton is shown in Table 2.

The average values of H’, d and D were 1.43, 0.30 and 2.09, respectively. The values of H’ and D were both within the range for normal diversity. In contrast, the value of d was indicated poor diversity, which may be due to the different calculation method for diversity index and the unrecovered evenness of zooplankton after the short-term EAS treatment^40, 41^.

In sum, the EAS operation dramatically removed the COD, TN, and TP from the eutrophic water body, indicating its efficient removal of contaminant. The algae blooms rapidly disappeared, showing EAS effects on eutrophication control. The abundance of typical eutrophic freshwater bacteria (*Flavobacterium* and *Pseudomonas*) decreased and the diversity of microbial community increased, indicating that the eutrophic freshwater became healthier. The diversity indices of phytoplankton and zooplankton indicated normal diversity of these species compared to eutrophic freshwater. *Chlorella* and *Chlamydomonas* were undetectable, further suggesting that the eutrophication was inhibited. It is worth noted that the concentration of TN and TP decreased in water and sediments as well as in suspended matters (indicated by the decrease of turbidity). The loss of TN is probably because of two reasons. One is that denitrification converted nitrate to N_2_, leaching nitrogen to atmosphere. The other is that living organism (e.g., microorganism) assimilated more ammonia for growth. The loss of TP is also possible because that living organism assimilated more phosphorus for growth. The mass balance of TN and TP is critical for understanding the underlying mechanism of EAS and thus should be conducted in the future. Based on these findings, we hypothesize that the microbial community plays the fundamental role in trophic levels. EAS promoted that microbial community and reconstructed the food web based on newly formed trophic relationships, which is key to the regulation of a healthy water ecosystem^42^. This bottom-up approach for the restoration of trophic levels is dependent on microbial community restoration. Subsequently, the phytoplankton and zooplankton community was restored, reflected by the healthy value of the diversity indices.

## Methods

### Operation process of EAS

During the 24 h feeding period, the lake water was pumped into the reactor at a rate of 0.075 m^3^/h, with a hydraulic retention time (HRT) of 8 h. During the formal operation stage, the influent and effluent flow was kept at 0.1 m^3^/h. The internally recycled water was moved to the influent. The 2 streams of water were completely mixed at the inlet of the self-priming pump. The inner ratio was 100% and the hydraulic cycle of the entire system was maintained by the self-priming pump. DO concentration was maintained at 3.5 ± 0.5 mg/L in the aerobic stage using an online, real-time control device.

### Test water body and sampling

The test water body is located at 121.539579°–121.539983°E, 31.314243°–31.314806°N, Shanghai, China (Fig. S2, the sketch map was create by Microsoft Software PowerPoint 2013, https://microsoft-powerpoint.en.softonic.com/; the latitude and longitude data: Google, DigitalGlobe, https://maps.google.com/). The total area of the water body is 8000 m^2^, and the water depth is 2–3 m. The main pollution sources were surface runoff and uncontrolled discharge of domestic wastewater due to the combined sewer. The water surface was initially covered by suspended algae clouds and was blackish green.

Four sampling points (1–4) were set up (Fig. S2) and water quality, sediment level, microbial community diversity, phytoplankton diversity, and zooplankton diversity were evaluated.

### Sampling for water quality and microbial community analysis

Samples for water quality analysis were collected every 3–5 d with HQM-1 sampling bottles (5 L) at 0.5 m below the surface. After 30 d of EAS operation, the water quality was greatly improved greatly and remained stable. Therefore, the samples for microbial community analysis were collected prior to initiation of EAS operation and after 30 d of EAS operation. A 1 L quantity of water was filtered through a 0.22 µm hydrophilic membrane (Millipore, USA) for water quality analysis. A 100 mL sample of water was filtered through a sterilized 0.22-μm filter (Millipore, USA) for microbial community analysis. To reflect the whole variation process of the restoration of the water body, the physiochemical parameters of water was constantly monitored for 90 days.

### Sediment sampling

Sediment samples were collected from the upper layer (0–20 cm depth) using stainless-steel sediment cores (40 cm long × 4 cm diam.), and stored in sterile 250-mL bottles. The sampling points were also set at positions marked from 1# to 4#. Within 3 h, the sediment cores were transported to the laboratory into a refrigerator and were then frozen and maintained at −20 °C until processing.

### Phytoplankton and zooplankton sampling

Phytoplankton and zooplankton were measured after the water quality had improved significantly (at 30 d of EAS operation) as additional evidence for the restored water ecosystem.

A plankton net with an aperture of 20 μm was used to collect the samples for qualitative analysis. By dragging the plankton net several times from the bottom to the surface of the lake the trapped phytoplankton were concentrated in the sampling net and then transformed into specimen bottles. A 4% wt./wt. formaldehyde solution was added to fix the individual plankton for further analysis. The samples for quantitative analysis were collected using HQM-1 sample bottles (5 L) at 0.5 m below the surface at each sampling point. A 1-L water sample for each sample point was aliquoted into sterile polyethylene bottles and then 15 mL of Lugol reagent was added. The samples were then transported in ice to the laboratory. The preparation procedures for using the Lugol reagent were done according to ref. 43.

A zooplankton net with a 20-μm aperture was used to obtain samples for qualitative analysis. By dragging the zooplankton net from the bottom to the surface of the lake several times, the trapped zooplankton were concentrated in the net and then transferred to specimen bottles. A 4% wt./wt. formaldehyde solution was added to fix the individual zooplankton for further analysis. The length of the sampling net was recorded.

### Analytical methods

#### Water quality

Water quality parameters (COD, TN, TP, NH_3_-N, NO_3_^−^-N, DO, turbidity, and chroma) were determined according to Standard Methods: 2540 D (Environmental Protection Agency, USA).

#### Sediment analysis

The moisture content and volatile solid (VS) content of sediment obtained after centrifugation were then measured by the gravimetric method. TN and TP of sediment samples were measured using the alkaline potassium persulfate digestion-UV spectrophotometric method and ammonium molybdate spectrophotometric method according to the Chinese national standard (CJ/T221-2005), respectively.

#### Microbial community analysis

Bacterial community structure of the initial raw water and water after 30 d EAS treatment were analyzed via MiSeq Illumina sequencing using the adaptor primers 338 F: (ACTCCTACGGGAGGCAGCA) and 806 R: (GGACTACHVGGGTWTCTAAT) for the V3-V4 regions of the 16S rRNA gene. DNA extraction and polymerase chain reaction (PCR) amplification was used based on a previously published method^44^. Equal quantities of the 3 PCR products per sample were pooled and purified with a QIAquick PCR Purification Kit (Qiagen, USA). Finally, a mixture of the amplicons from the different samples was sent to the MiSeq Illumina platform at Suzhou Jinweizhi Bio-pharm Biotechnology Co., Ltd. (Suzhou, China) for sequencing. The treatment of the raw sequences is detailed in the Supporting Information.

#### Phytoplankton analysis

Each 1 L water sample for the quantitative analysis of phytoplankton was concentrated to 70 mL through repeated sedimentations. A microscope (Nikon 80i, Nikon Co. Japan) was used to identify the phytoplankton species based on micro topography. Qualitative analysis was repeatedly conducted until no new species were found. The density of phytoplankton in a 0.25-mL concentrated water sample was counted using a dilute plate method under microscope. Density determinations were made 3 times.

The concentration of phytoplankton = the total number counted in 0.25 mL water sample × 70 mL/(0.25 mL × 1 L)

*Units: concentration* (*cell*/*L*), *the total cell count*)

#### Zooplankton analysis

A microscope (Nikon 80i, Nikon Co. Japan) was used to identify the species of zooplankton based on the micro topography. Qualitative analysis was repeatedly conducted until no new species were found. The total number of the collected zooplanktons was counted using dilute plate method under microscope with 3 replicates.

The concentration of zooplanktons = the total counted number of collected zooplanktons/(the sectional area of sampling net × the recorded length of sampling net)

*Units: concentration (ind/m* ^*3*^ *), the total counted number (ind), the sectional area of sampling net (m* ^*2*^ *), the recorded length of sampling net (m)*

#### Phytoplankton and zooplankton diversity analysis

The richness of phytoplankton and zooplankton community was evaluated using the Shannon-Wiener diversity index (H′), Margalef richness index (d), and Simpson index of species similarity (D). The calculation methods for above 3 indexes are listed as follows:

Calculation of diversity index

Shannon-Wiener diversity index:

1 $$H^{\prime} =-\sum [(\frac{{n}_{i}}{n})\times lo{g}_{2}(\frac{{n}_{i}}{n})]$$

where n_i is the density of species i, and n is the total density gravity. The degree of diversity was calculated as follows:

H′ > 3.5, high diversity;

H′ = 2.5–3.5, diverse;

H′ = 2.0–2.5, good diversity; H′ = 1.0–2.0, normal diversity; H′ < 1.0, poor diversity.

Margalef richness index:

2 $$d=\frac{S-1}{lo{g}_{2}N}$$

where S is the number of species, and N is the total number of individuals found. The degree of richness was ranked and calculated as follows:

d > 3.5, high diversity;

d = 2.5–3.5, diverse; d = 2.0–2.5, good diversity; d = 1.0–2.0, normal diversity; d < 1.0, poor diversity.

Simpson index of species similarity:

3 $$D=\frac{1}{{\sum ({n}_{i}/N)}^{2}}$$

where n_i_ is the density of species i, and N is the total number of individuals found.

The degree of richness was ranked and calculated as follows:

D > 6, high diversity;

D = 3.0–6.0, good diversity; D = 2.0–3.0, normal diversity;

D < 2.0, poor diversity.

## Electronic supplementary material

Supporting Info
