# Supplementary material for: Ecosystem activation system (EAS) technology for remediation of eutrophic freshwater
Source: Sci Rep. 2017 Jul 6;7:4818. doi: 10.1038/s41598-017-04306-3 (PMC5500588; doi:10.1038/s41598-017-04306-3)
Supplement: Supplementary file 1 — Supporting Info [file 41598_2017_4306_MOESM1_ESM.pdf]

Supporting Information of "Ecosystem activation system (EAS) technology for remediation of eutrophic freshwater"

Xiaoli Chai[1], Boran Wu[1], Zhongshuo Xu[1], Ning Yang[1], Liyan Song[2], Jingjing Mai[1], Yang Chen[1], Xiaohu Dai[1,*]

[1]State Key Laboratory of Pollution Control and Resource Reuse, College of Environmental Science and Engineering, Tongji University, 1239 Siping Road, Shanghai, 200092, China

[2]Environmental Microbiology and Ecology Research Center, Chongqing Institute of Green and Intelligent Technology, Chinese Academy of Science (CAS), 266 Fangzheng Avenue, Chongqing, 400714, China

*corresponding author

Number of pages: 9

Number of table: 4

Number of figures: 2

Contents:

Page S2 Raw sequences treatment

Page S3 Table S1 Water quality of the test water body

Page S4 Table S2 Concentration of TP and TN in the sediment (wt. % DS)

Page S5 Table S3 The number concentration of phytoplankton /$10^4 \cdot L^{-1}$

Page S6 Table S4 Species of phytoplankton

Page S7 Figure S1 Sensory quality of the test water body (a) sensory quality index (b) photo of raw water body (c) photo water body after 15 days' operation of the EAS (d) advanced plants growing on the underwater rocks after 30 days

Page S8 Figure S2 Location of the test water body

Page S9 Reference

Raw sequences treatment

High-quality V3-V4 tags from raw paired-end reads was acquired by Trimmomatic (http://www.usadellab.org/cms/index.php?page=trimmomatic)[1]. The rules are: the maximum number of errors in the barcode was 0; the maximum length of homopolymer run was 6; the number of mismatches in the primer was 2. The reads with more than 10% of bases with a quality score of Q < 20, ambiguous and unassigned characters and adapter contamination were removed. Chimeras from high-quality tags was checked by UCHIME (http://drive5.com/uchime)[2]. Operational taxonomic units (OTUs) at 97% identity was classified by Usearch (http://www.drive5.com/usearch/)[3,4] using furthest neighbor clustering. Representative sequences of OTUs were aligned to the SILVER (version: SSU119 http://www.arb-silva.de/) for bacteria 16S rRNA genes. Bacterial diversity index (Shannon, Simpson, Ace, Chao1, and coverage) was calculated by Mothur (version v.1.30.1). The sequences were then phylogenetically assigned to taxonomic classifications using an RDP-naïve Bayesian rRNA classifier with a confidence threshold of 70%[5].

Table S1 Water quality of the test water body

	TN/(mg/L)	TP/(mg/L)	COD/(mg/L)	DO/(mg/L)	pH
raw	1.12±0.20	0.28±0.02	60.88±2.54	-	7.34±0.37
90d	0.86±0.13	0.05±0.03	13.52±1.80	5.0±0.4	7.55±0.25
Chinese standard Class II	≤0.5	≤0.1	≤15	≥6	6–9
Chinese standard Class III	≤1.0	≤0.2	≤20	≥5	6–9

Table S2 Concentration of TP and TN in the sediment (wt. % DS)

	TN	TP	VS
Raw water	0.230 ± 0.009	0.366 ± 0.071	3.78 ± 0.27
90 days' treatment	0.127 ± 0.009	0.209 ± 0.071	2.87 ± 0.16

Table S3 Number concentration of phytoplankton /10^4 L^{-1}

	**Max**	**Min**	**Average**
Cryptophyta	96.28	53.00	74.30
Chlorophyta	74.76	41.77	60.92
Bacillariophyta	15.19	9.64	12.22
Xanthophyceae	0.97	0.47	0.72
Euglenophyta	3.67	0.52	1.68
Pyrrophyta	0.04	--*	0.02
Chrysophyta	0.03	--	0.01
Total	179.07	126.32	149.87

*Note: --undetected

Table S4 Species of phytoplankton

Dominant species	Mcnaughton index*	Concentration (10^4 L^{-1})	Occurring frequency (%)
Chroomonas acuta	0.411	61.56	100
Pediastrum simplex v. *duodenarium*	0.341	51.18	100
Cryptomonas ovata	0.057	8.49	100
Nitzschia sp.	0.055	8.24	100
Scenedesmus quadricauda	0.030	4.54	100
Cry. erosa	0.028	4.25	100

* Mcnaughton index, $Y = \frac{n_i}{N} \times f_i$, where n_i is the density of specie i, and N is the total number of individuals found and f_i is the occurrence frequency of specie i at the sampling points. If Y>0.02, the specie is identified as the dominant specie.

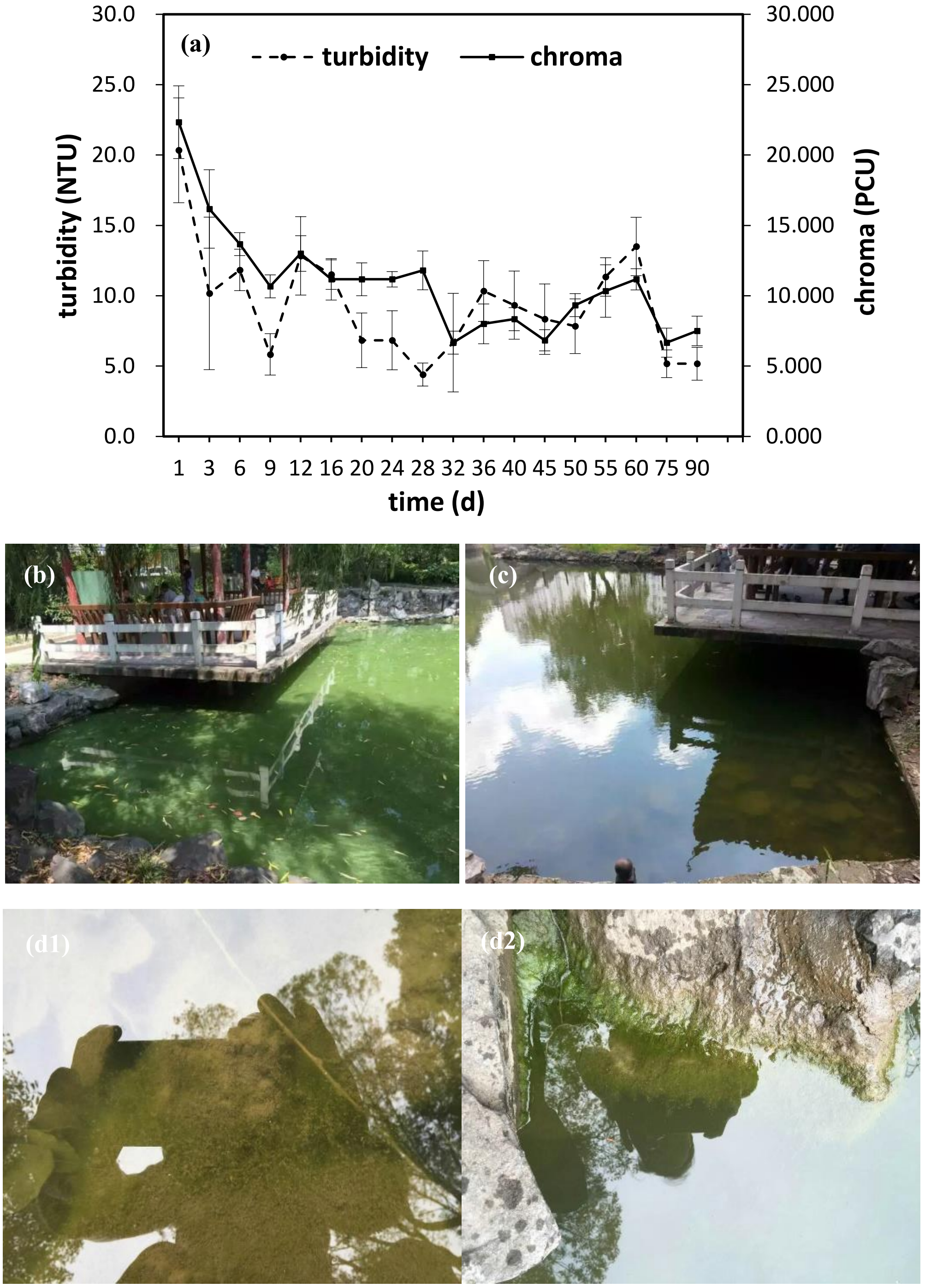

Figure S1 Sensory quality of the test water body

(a) sensory quality index (b) photo of raw water body (c) photo water body after 15 days' operation of the EAS (d1~2) advanced plants growing on the underwater rocks after 30 days

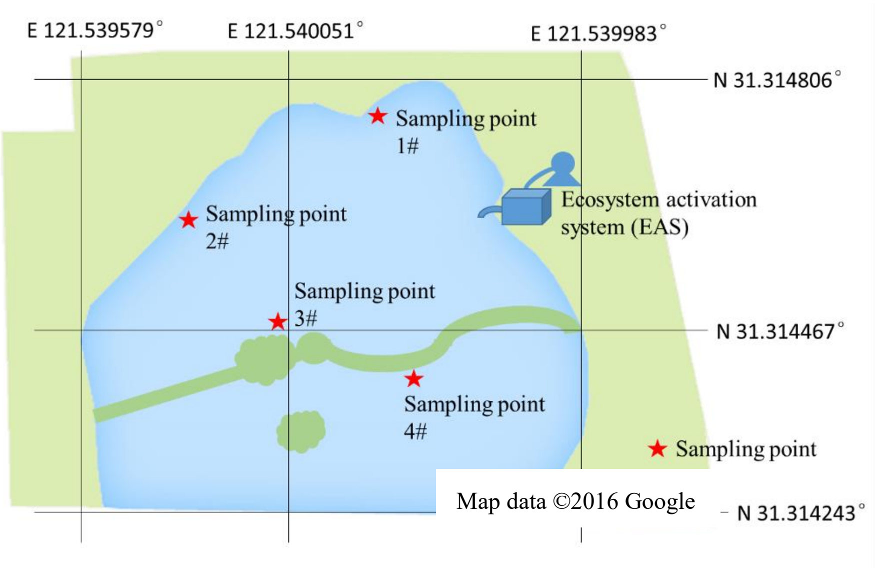

Figure S2 Location of the test water body. The sketch map was create by Microsoft Software PowerPoint 2013 (https://microsoft-powerpoint.en.softonic.com/); the latitude and longitude data: Google, DigitalGlobe (https://maps.google.com/).

References

1 Bolger, A. M., Lohse, M. & Usadel, B. Trimmomatic: a flexible trimmer for Illumina sequence data. Bioinformatics, **30**, 2114-2120 (2014).

2 Edgar, R. C., Haas, B. J., Clemente, J. C., Quince, C. & Knight, R. UCHIME improves sensitivity and speed of chimera detection. Bioinformatics **27**, 2194-2200 (2011).

3 Edgar, R. C. Search and clustering orders of magnitude faster than BLAST. Bioinformatics **26**, 2460-2461 (2010).

4 Edgar, R. C. UPARSE: highly accurate OTU sequences from microbial amplicon reads. Nat. methods **10**, 996-998 (2013).

5 Wang, Q., Garrity, G. M., Tiedje, J. M. & Cole, J. R. Naive Bayesian classifier for rapid assignment of rRNA sequences into the new bacterial taxonomy. Appl. Environ. Microbiol. **73**, 5261-5267 (2007).
